# Effect of rhBMP-2 Immobilized Anorganic Bovine Bone Matrix on Bone Regeneration

**DOI:** 10.3390/ijms160716034

**Published:** 2015-07-14

**Authors:** Jung-Bo Huh, June-Jip Yang, Kyung-Hee Choi, Ji Hyeon Bae, Jeong-Yeol Lee, Sung-Eun Kim, Sang-Wan Shin

**Affiliations:** 1 Department of Prosthodontics, Dental Research Institute, Biomedical Research Institute, Institute of Translational Dental Sciences, School of Dentistry, Pusan National University, YangSan 676-870, Korea; E-Mails: huhjb@pusan.ac.kr (J.-B.H.); bjhyeon@gmail.com (J.H.B.); 2 Advanced Prosthodontics, Postgraduate School of Clinical Dentistry, Institute for Clinical Dental Research, Korea University, Seoul 152-703, Korea; E-Mails: gumcab@hanmail.net (J.-J.Y.); wddc@korea.ac.kr (J.-Y.L.); 3 Research Development Institute, Cowellmedi Co., Ltd., Busan 617-801, Korea; E-Mail: ckh@cowellmedi.com; 4 Department of Orthopedic Surgery & Rare Diseases Institute, Korea University Medical Center, Guro Hospital, Seoul 152-703, Korea

**Keywords:** bone regeneration, anorganic bovine bone, Bio-Oss^®^, rhBMP-2

## Abstract

Anorganic bovine bone matrix (Bio-Oss^®^) has been used for a long time for bone graft regeneration, but has poor osteoinductive capability. The use of recombinant human bone morphogenetic protein-2 (rhBMP-2) has been suggested to overcome this limitation of Bio-Oss^®^. In the present study, heparin-mediated rhBMP-2 was combined with Bio-Oss^®^ in animal experiments to investigate bone formation performance; heparin was used to control rhBMP-2 release. Two calvarial defects (8 mm diameter) were formed in a white rabbit model and then implanted or not (controls) with Bio-Oss^®^ or BMP-2/Bio-Oss^®^. The Bio-Oss^®^ and BMP-2/Bio-Oss^®^ groups had significantly greater new bone areas (expressed as percentages of augmented areas) than the non-implanted controls at four and eight weeks after surgery, and the BMP-2/Bio-Oss^®^ group (16.50 ± 2.87 (*n* = 6)) had significantly greater new bone areas than the Bio-Oss^®^ group (9.43 ± 3.73 (*n* = 6)) at four weeks. These findings suggest that rhBMP-2 treated heparinized Bio-Oss^®^ markedly enhances bone regeneration.

## 1. Introduction

For dental implant treatment, primary surgical augmentations are often required due to the lack of alveolar bone at surgical sites [1]. The purpose of this procedure is to obtain sufficient vertical and horizontal volumes of alveolar bone. Currently, alveolar bone augmentation by autogenous block bone grafting is recognized as a gold standard [2]. However, amounts of block autogenous bone graft are limited at donor site, and an additional bone graft donor procedure is required, which increases the impact of surgery and introduces the risk of postoperative complications, such as, infection, bleeding, pain, edema, and damage to nerves and blood vessels [3]. As an alternative, various bone substitutes are currently used [4]. Bio-Oss^®^ (Geistlich, Wolhusen, Switzerland) is a deproteinized bovine bone comprised of calcium-deprived carbonate apatite with the same mineral content as human bone [5]. This material has been used for a considerable time in oromaxillofacial fields for bone grafting, regenerating defect areas, and for periodontal regeneration [5]. Due to the very low absorption rate of Bio-Oss^®^ and its trabecular structure, which helps introduce blood cells and bone cells, new bone deposition can be accelerated. This means that the material has the features of a scaffold with excellent biocompatibility [6]. Clinical studies conducted during the last decade have reported the utility of Bio-Oss^®^ as a biocompatible material for the regeneration of intra-oral bone loss [7]. However, according to experimental studies, osteoblast proliferation and differentiation are known to be lower for Bio-Oss^®^ than for autogenous bone [8].

To overcome this limitation, trials have been conducted to combine an osteoconductive scaffold with an osteoinductive protein to enhance bone regeneration performance. Osteoinductive proteins, such as, recombinant human bone morphogenetic protein-2 (rhBMP-2) cause the differentiations of mesenchymal stem cells and pre-osteoblasts to osteoblasts, and promote the migration of osteoblastic cells [9,10]. In recent clinical and histological studies, the addition of rhBMP-2 during the bone graft process resulted in excellent performance [11]. In other studies, the addition of rhBMP-2 protein was reported to have induced orthotropic and ectopic bone formation [12]. In terms of its interaction with the immune system, the risks posed by rhBMP-2 have been confirmed to be low [13]. Furthermore, the bone regeneration ability of rhBMP-2 is enhanced by carrier materials [14,15], which can be easily applied, secure a space for regeneration, and are bio-absorbable and biodegradable. Collagen is the only Food and Drug Administration (FDA)-approved material for rhBMP-2 delivery, but lacks osteoconductivity and has poor mechanical features [16]. According to previous studies on the use of a heterogeneous bone graft as a carrier, space was sufficiently secured and the graft was found to be an excellent carrier of osteoinductive proteins [15]. The combined use of a bone graft materials and rhBMP-2 promotes mature bone regeneration, because rhBMP-2 has the potential to improve the performance of the bone regeneration procedure [11]. So, we considered the use of rhBMP-2 might overcome the limitations of Bio-Oss^®^, which has poor osteoinductive ability.

Despite the excellent effects of rhBMP-2 on bone regeneration, it has been reported to have several limitations, which include a high cost, the need for a large amount, and its short *in vivo* half-life [17]. Many carriers for rhBMP-2 have been studied, but all have failed to exhibit adequate control of rhBMP-2 release [18]. In this study, rhBMP-2 release was controlled using heparin that is a sulfated natural simple polysaccharide with affinity for many growth factors [19]. In previous studies, when rhBMP-2 and heparin were combined on the surface of titanium, rhBMP-2 release was well controlled, and consequently, the anti-inflammatory effects and the functions of osteoinductive cells were enhanced. In an *in vitro* study on a demineralized bone matrix sequentially treated with heparin and rhBMP-2, Alkaline phosphatase (ALP) activity and calcium accumulation in tissues were enhanced [20]. Therefore, in this study, heparin/rhBMP-2 treated Bio-Oss^®^ (the BMP-2/Bio-Oss^®^ group) was compared with non-implanted controls and Bio-Oss^®^ (the Bio-Oss^®^ group) with respect to new bone formation in a calvarial defect rabbit model.

## 2. Results

### 2.1. Surface Morphology of Bio-Oss^®^

**Figure 1:**
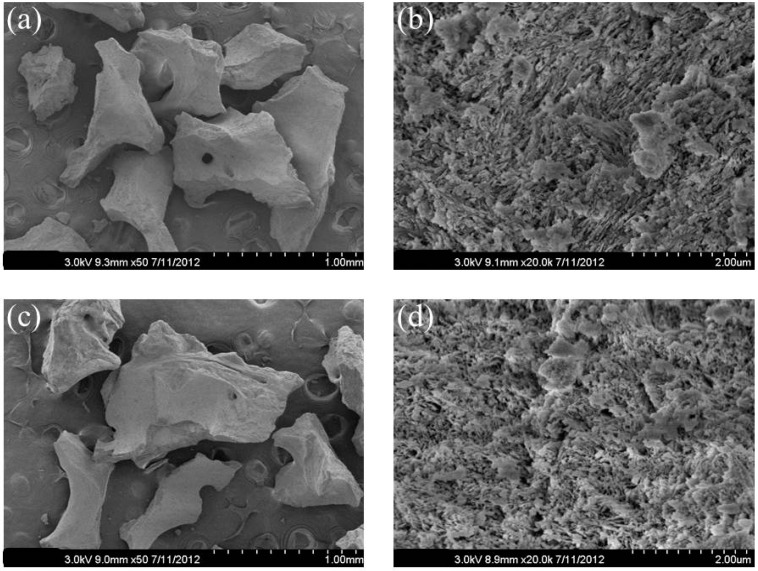

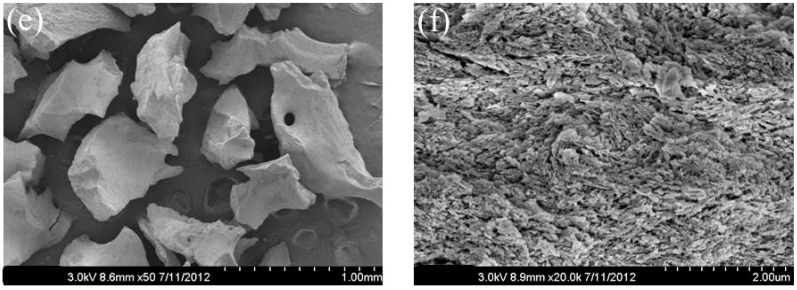
Scanning electron microscopy (SEM) of (**a**) Bio-Oss^®^; (**c**) heparinized Bio-Oss^®^; (**e**) heparinized rhBMP-2-Bio-Oss^®^ (original magnification ×50); higher magnification images (×20,000) of (**b**) Bio-Oss^®^; (**d**) heparinized Bio-Oss^®^; and (**f**) heparinized rhBMP-2-Bio-Oss^®^.

The surfaces morphologies of Bio-Oss^®^, heparinized Bio-Oss^®^ and heparinized rhBMP-2-Bio-Oss^®^ (BMP-2/Bio-Oss^®^ group) were compared by SEM to observe their microstructures (Figure 1), and were found to be similar.

**Figure 2:**
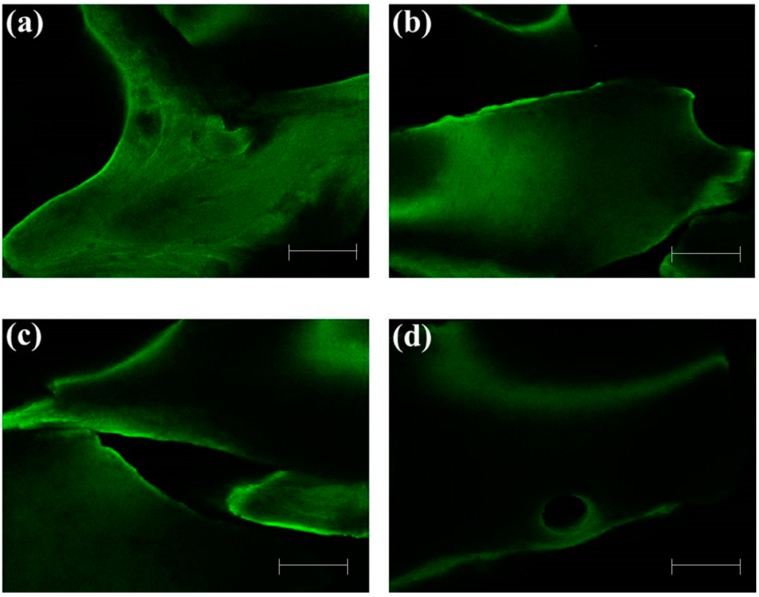
Confocal laser scanning microscopy (CLSM) images of GFP-conjugated rhBMP-2-Bio-Oss^®^ surfaces for (**a**) 1 day; (**b**) 3 days; (**c**) 7 days; and (**d**) 10 days in 0.1 M MES buffer. Scale Bars: 0.5 mm.

In addition, we confirmed that green fluorescent protein (GFP)-conjugated rhBMP-2 was immobilized on heparinized Bio-Oss^®^ surfaces. As shown in Figure 2, GFP-conjugated rhBMP-2 was observed on heparinized Bio-Oss^®^ surfaces for 10 days in 0.1 M 2-(*N*-morpholino)ethanesulfonic acid (MES) buffer.

### 2.2. Surface Composition Assay

**Figure 3:**
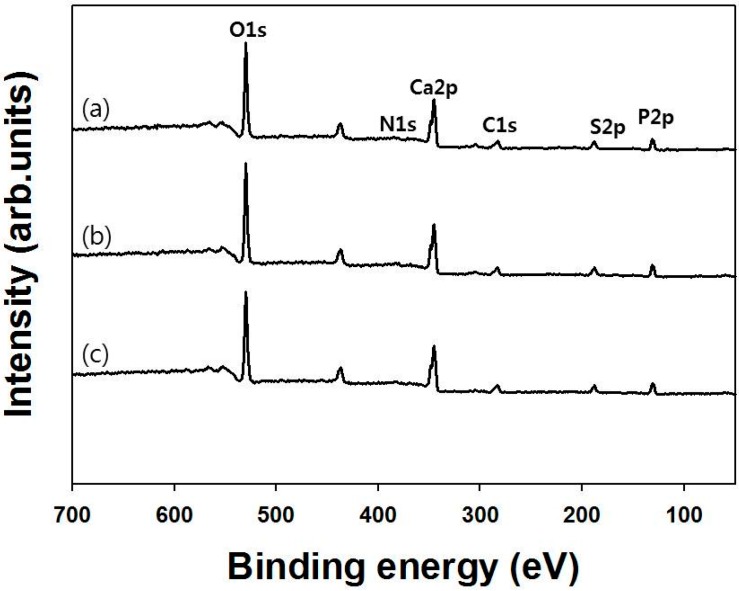
X-ray photoelectron spectroscopy wide-scan spectra of (a) Bio-Oss^®^; (b) heparinized Bio-Oss^®^; and (c) heparinized rhBMP-2-Bio-Oss^®^.

The X-ray photoelectron spectroscopy (XPS) wide-scan spectra of Bio-Oss^®^, heparinized Bio-Oss^®^ and heparinized rhBMP-2-Bio-Oss^®^ (BMP-2/Bio-Oss^®^ group), and their surface element chemical composition are shown in Figure 3 and Table 1, respectively. Successful heparin modification of Bio-Oss^®^ was confirmed by a 1.23% increase in N content and a 1.12% increase in S content *versus* Bio-Oss^®^. Furthermore, XPS confirmed the immobilization of rhBMP-2 on heparinized Bio-Oss^®^ surfaces by showing increases in N content and decreases in S content *versus* heparinized Bio-Oss^®^.

**Table 1:** Surface elemental compositions of Bio-Oss^®^, heparinized Bio-Oss^®^, and heparinized rhBMP-2-Bio-Oss^®^.

Substrate	Ca%	C%	N%	O%	P%	S%
Bio-Oss^®^	18.55	16.96	–	51.38	12.59	0.52
Heparinized Bio-Oss^®^	18.29	14.11	1.23	52.95	11.78	1.64
Heparinized rhBMP-2- Bio-Oss^®^ (BMP-2/Bio-Oss^®^)	18.07	14.26	2.25	52.63	12.03	0.76

### 2.3. Release of rhBMP-2

The *in vitro* release of rhBMP-2 from BMP-2/Bio-Oss^®^ was evaluated for up to 28 days. At day 1, the amount of rhBMP-2 released was 37.3% ± 5.15% for BMP-2/Bio-Oss^®^. Over the 28-day study period, the amount of rhBMP-2 released was 79.41% ± 4.65%.

**Figure 4:**
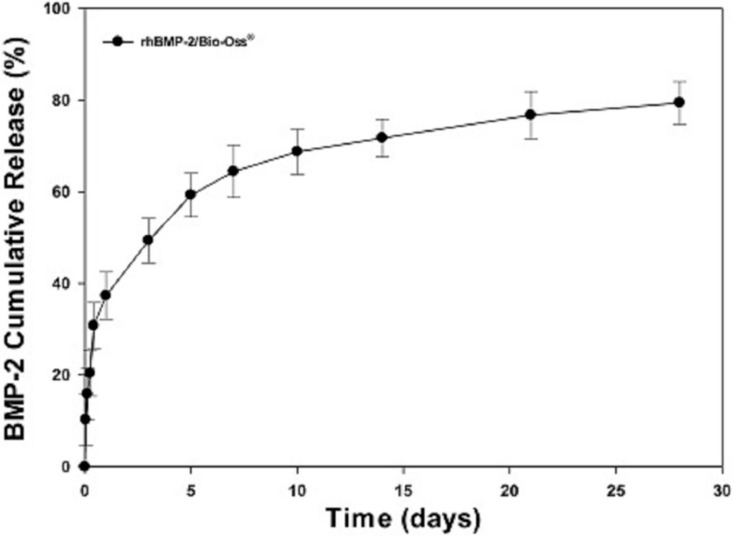
The *in vitro* release of rhBMP-2 from BMP-2/Bio-Oss^®^ group.

### 2.4. Micro-Computed Tomography (μCT)

**Figure 5:**
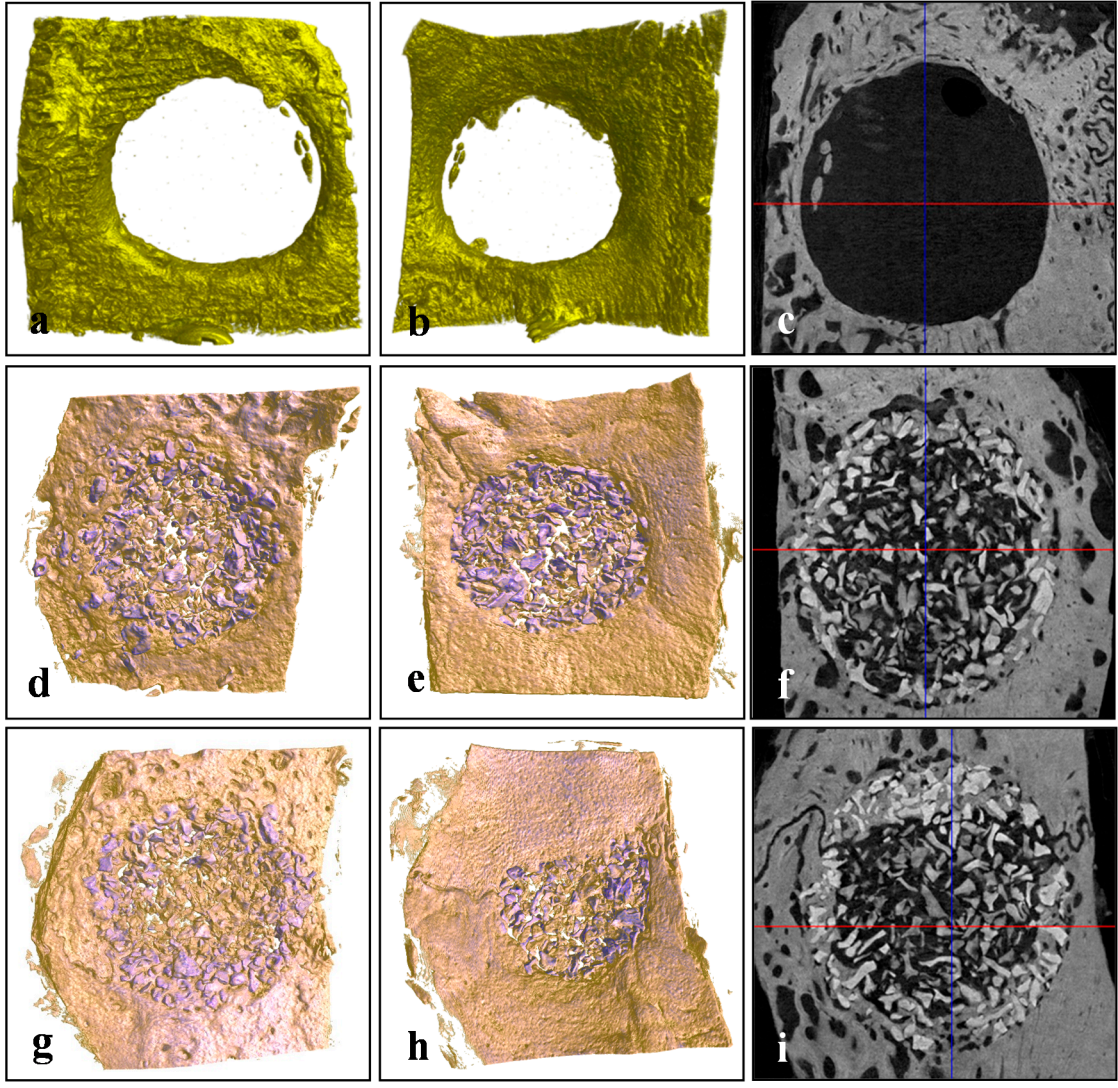
Representative μCT images of each group at four weeks after surgery. (**a**–**c**) controls; (**d**–**f**) the Bio-Oss^®^ group; (**g**–**i**) the BMP-2/Bio-Oss^®^ group; (**a**,**d**,**g**) outer images of defect sites; (**b**,**e**,**h**) inner images of defect sites; (**c**,**f**,**i**) horizontally sectioned images of defect sites; remained Bio-Oss^®^ particles are purple colored.

**Figure 6:**
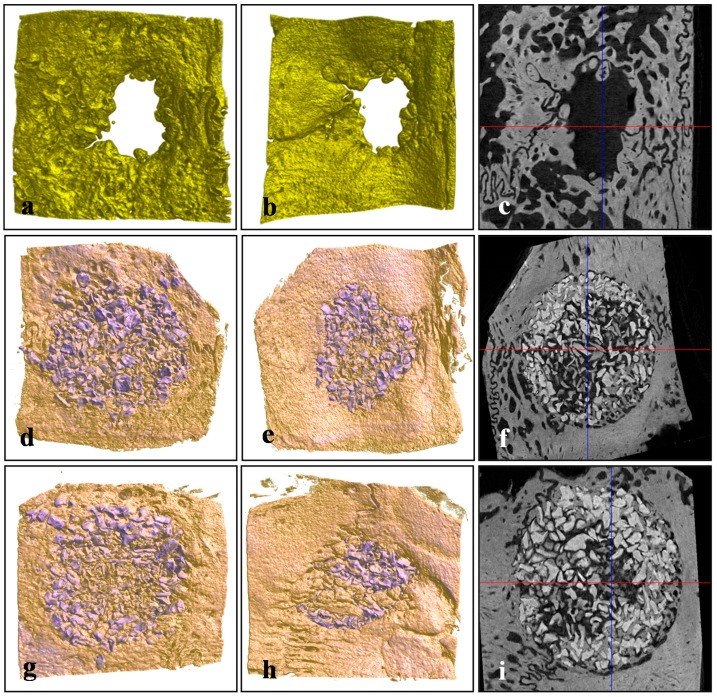
Representative μCT images at eight weeks after surgery. (**a**–**c**) controls; (**d**–**f**) Bio-Oss^®^ group; (**g**–**i**) BMP-2/Bio-Oss^®^ group; (**a**,**d**,**g**) outer images of defect sites; (**b**,**e**,**h**) inner images of defect sites; (**c**,**f**,**i**) horizontally sectioned images of defect sites; Bio-Oss^®^ particles are purple colored.

Total volumes of newly formed bone within regions of interest (ROIs) were measured by assigning a threshold for total bone content (including trabecular and cortical bone). The Bio-Oss^®^ and BMP-2/Bio-Oss^®^ groups had significantly greater new bone area than the non-implanted controls at four and eight weeks after surgery (*p* < 0.05), and the BMP-2/Bio-Oss^®^ group had significantly greater new bone area than the Bio-Oss^®^ group at four and eight weeks (*p* < 0.05). Tissue areas in the Bio-Oss^®^ and BMP-2/Bio-Oss^®^ groups were also significantly greater than in the non-implanted control group at four and eight weeks (*p* < 0.05). Two-way ANOVA revealed that rhBMP-2 treatment had a strong influence on new bone formation (*p* < 0.05) (Table 2, Figure 5 and Figure 6).

**Table 2:** The total volumes (%) of newly formed bone in the control, Bio-Oss^®^, and rhBMP-2 groups (means ± SD (number of specimens)).

Group	4 Weeks	8 Weeks	*p*-Value
Control	4.06 ± 1.37 (6)	8.12 ± 2.11 ^**‡**^ (6)	0.003
Bio-Oss^®^	10.86 ± 1.54 * (6)	17.90 ± 2.50 *^,^^**‡**^ (6)	0.000
BMP-2/Bio-Oss^®^	21.21 ± 4.28 *^,**†**^ (6)	32.03 ± 4.54 *^,^^**†**,**‡**^ (6)	0.002
*p*-value	0.011	0.000	–

* Significant different from controls (*p* < 0.05); ^**†**^ Significant different from the Bio-Oss^®^ group (*p* < 0.05); ^**‡**^ Significantly different at four and eight weeks after surgery (*p* < 0.05).

### 2.5. Histological Observations

**Figure 7:**
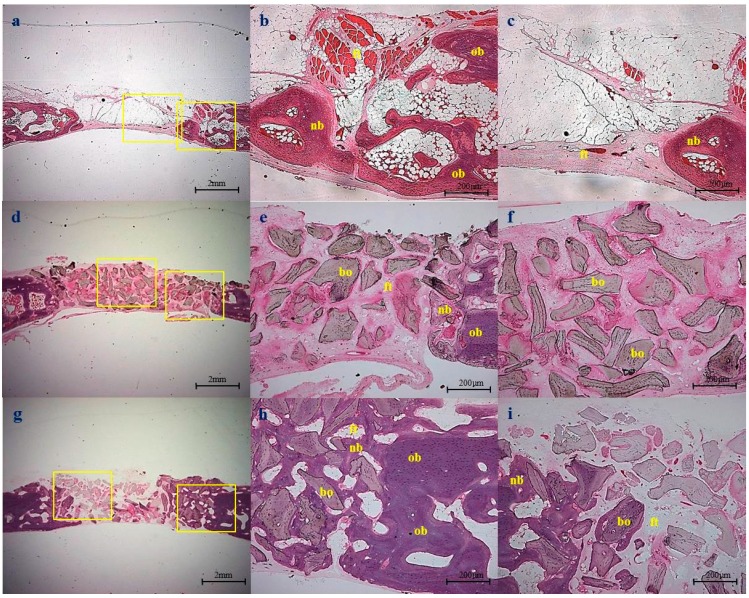
Histologic sections obtained at four weeks after surgery: (**a**–**c**) The non-implanted control group, showing new bone (nb) formation from the cut edge; (**d**–**f**) Bio-Oss^®^ group; (**g**–**i**) BMP-2/Bio-Oss^®^ group. New bone was of better quality than in the Bio-Oss^®^ group. Original magnifications were ×12.5 for (**a**,**d**,**g**) and ×40 for the others. (nb: new bone, ft: fibrovascular tissue, bo: Bio-Oss^®^ particle, ob: old bone). The enlarged pictures of the yellow boxes are in the middle and right part.

**Figure 8:**
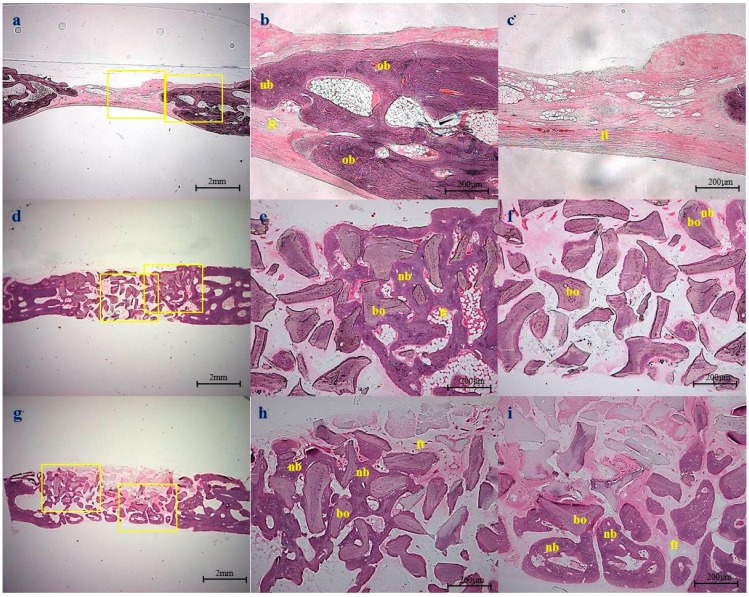
Histologic sections at eight weeks after surgery: (**a**–**c**) non-implanted control group, showing new bone (nb) formation from the cut edge; (**d**–**f**) Bio-Oss^®^ group. New bone formation was observed between particles, but no new bone was observed in the middle portions of defects; (**g**–**i**) BMP-2/Bio-Oss^®^ group. New bone was of greater quality than in the Bio-Oss^®^ group. Original magnifications were ×12.5 for (**a**,**d**,**g**) and ×40 for the others. (nb: new bone, ft: fibrovascular tissue, bo: Bio-Oss^®^ particle, ob: old bone).

Control group: At four weeks after surgery, minimal amounts of new bone tissue had formed from the defect margins towards the central portion. Most of the defects were filled with a thin layer of fibrous connective tissue, which did not attain the thickness of native calvarial bone (Figure 7). At eight weeks, bone maturation had increased to some extent at the periphery of defect margins (Figure 8).

Bio-Oss^®^ group: At four weeks after surgery, Bio-Oss^®^ particles were well maintained under a connective tissue layer. New bone was seen at the periphery of defect margins and around Bio-Oss^®^ particles. Immature bone tissue was not only interconnected, but also partially enclosed Bio-Oss^®^ particles (Figure 7e). At eight weeks, no resorption of graft materials was noticed, but bone formation was greater than that observed at four weeks. Furthermore, bony tissues around Bio-Oss^®^ particles were more interconnected than at four weeks (Figure 8e).

BMP-2/Bio-Oss^®^ group: At four weeks after surgery, a great amount of new bone formation was observed. The newly formed bone was of the lamellar type and had been deposited directly onto the Bio-Oss^®^, and extended randomly to form an anastomosed network of trabeculae (Figure 7h,i). At eight weeks, the quantity of the newly formed bone improved further, and specimens exhibited more advanced stages of remodeling and consolidation. Other findings in new bone included lines separating from more recently deposited bone, concentric rings of the Haversian system, and fatty marrow (Figure 8h,i).

### 2.6. Histometric Analysis

The defects were partially closed in the Bio-Oss^®^ and BMP-2/Bio-Oss^®^ groups at each time point, but the Bio-Oss^®^ and BMP-2/Bio-Oss^®^ groups showed significantly greater defect closure than the control group at four and 8eight weeks (*p* < 0.05, Table 3). In particular, the BMP-2/Bio-Oss^®^ group showed significantly greater defect closure than the Bio-Oss^®^ group at eight weeks (*p* < 0.05, Table 3). In terms of percentages of new bone in augmented areas, the Bio-Oss^®^ and BMP-2/Bio-Oss^®^ groups had significantly greater new bone area than non-implanted controls at four and eight weeks (*p* < 0.05, Table 4), and the BMP-2/Bio-Oss^®^ group had significantly greater new bone area than the Bio-Oss^®^ group at four weeks (*p* < 0.05, Table 4). Two-way ANOVA revealed that rhBMP-2 treatment had a strong influence on histometric parameters (*p* < 0.05).

**Table 3:** Percentages of the defect closure (means ± SD (number of specimens)).

Group	4 Weeks	8 Weeks	*p*-Value
Control	7.83 ± 3.92 (6)	13.82 ± 3.21 ^**‡**^ (6)	0.016
Bio-Oss^®^	20.91 ± 6.40 * (6)	38.52 ± 5.01 *^,**‡**^ (6)	0.000
BMP-2/Bio-Oss^®^	29.28 ± 6.29 *^,^^**†**^ (6)	58.67 ± 7.95 *^,**†**,**‡**^ (6)	0.000
*p*-value	0.002	0.000	

* Significantly different from non-implanted controls (*p* < 0.05); ^**†**^ Significantly different from the Bio-Oss^®^ group (*p* < 0.05); ^**‡**^ Significantly different at four and eight weeks after surgery (*p* < 0.05).

**Table 4:** Amounts of new bone as percentages of augmented areas at four and eight weeks after surgery (means ± SD (number of specimens)).

Group	4 Weeks	8 Weeks	*p*-Value
Control	4.13 ± 2.50 (6)	7.49 ± 1.92 ^**‡**^ (6)	0.026
Bio-Oss^®^	9.43 ± 3.34 * (6)	17.76 ± 1.66 *^,**‡**^ (6)	0.000
BMP-2/Bio-Oss^®^	16.50 ± 2.57 *^,**†**^ (6)	29.20 ± 4.68 *^,^^**†**,**‡**^ (6)	0.000
*p*-value	0.000	0.000	

* Significantly different from non-implanted controls (*p* < 0.05); ^**†**^ Significantly different from the Bio-Oss^®^ group (*p* < 0.05); ^**‡**^ Significant different at 4 and 8 weeks after surgery (*p* < 0.05).

## 3. Discussion

In clinical studies conducted over the last decade, the utility of Bio-Oss^®^ as a biocompatible material in the field of intra-oral bone loss regeneration has been demonstrated [7]. However, Bio-Oss^®^ has a weaker osteo-inductive ability than other materials and poor osteoblast differentiation ability [21]. Previous animal studies confirmed various bone graft materials such as demineralized bone matrix, bovine deorganified crystalline bone matrix, and polymer scaffold are excellent carriers of osteo-inductive proteins [15]. In previous study, the combination of the Bio-Oss^®^ with rhBMP-2 enhanced the maturation of bone regeneration and could increase the graft to bone contact in humans [22]. Especially, Hänseler *et al.* [23] said that the surface of Bio-Oss^®^ is 1000-fold higher than HA/TCP, and Bio-Oss^®^ have a high affinity to rhBMP-2. This is the meaning that Bio-Oss^®^ is good material for delivery of rhBMP-2. Experiments on the use of bone grafts containing rhBMP-2 have reported significantly better bone regeneration outcomes, but the simple addition of rhBMP-2 to a bone graft results in the rapid loss of rhBMP-2. To overcome this problem, several different concepts of delivering rhBMP-2 have been studied. Polymerized natural collagen, which has been used for a long time as rhBMP-2 carrier, is the only FDA-approved material for rhBMP-2 delivery. However, collagen releases rhBMP-2 rapidly due to its low affinity, and at 14 days post-implantation only around 5% of the rhBMP-2 remains [24]. This initial massive release of rhBMP-2 differs completely from what actually happens in the body, as under natural conditions at bone fracture sites, rhBMP-2 levels gradually increase to peak at around 21 days post-injury [25]. Thus, it appears healing may be improved if rhBMP-2 release is similar to that observed during natural healing. For large defects that require a protracted healing time, the effective duration of rhBMP-2 release can be extended by controlling its release rate, and consequently, improve bone regeneration [26,27]. In this study on the combined application of rhBMP-2 and heparin for Bio-Oss^®^ bone grafting, heparin was used to control rhBMP-2 release. The rhBMP-2 was successfully released over a long time [28]. Heparin is known to have strong affinity for growth factors, and since rhBMP-2 contains a positively charged amino acid residue, it can interact with negative heparin [19,29]. In its presence, the half-life period of rhBMP-2 has been reported to be increased about 20-fold [29]. Systems that minimize rhBMP-2 release not only promote bone formation at treatment sites but also restrict unwanted pathological phenomena [30]. In many studies, the combination of rhBMP-2 and heparin has been shown to have an anti-inflammatory effect and promote osteoblast functions [31,32]. In this study, only BMP-2 coated Bio-Oss^®^ group was not included because, in our previous study [28], the release kinetics of rhBMP-2 were observed in the rhBMP-2-Bio-Oss and heparinized rhBMP-2-Bio-Oss, rhBMP-2 was rapidly released in the rhBMP-2-Bio-Oss group at an early stage. Meanwhile, more rhBMP-2 was released in the heparinized rhBMP-2-Bio-Oss group than in the rhBMP-2-Bio-Oss group. Furthermore, in heparinized rhBMP-2-Bio-Oss, rhBMP-2 was continuously released during the two-week period, and approximately 20% of the original amount of rhBMP-2 seemed to be released after the two-week period, showing a tendency of continuous release [28]. This drug delivery system using heparinized dopamine was applied in sequential delivery of BMP-2 and BMP-7 for bone regeneration using a heparinized collagen membrane [33] and improving osteoblast functions and bone formation upon growth factor immobilization on titanium modified with heparin [34,35]. Through these previous studies, it was concluded that rhBMP-2 immobilization is better than simple adhesion onto scaffold.

In this study, histological findings and Micro CT analysis revealed the Bio-Oss^®^ group and the BMP-2/Bio-Oss^®^ group showed significantly better bone regeneration outcomes than the non-implanted control group at four and eight weeks after surgery. At four and eight weeks, the BMP-2/Bio-Oss^®^ group showed significantly greater bone regeneration than the Bio-Oss^®^ group. This was presumably because eventual bone regeneration outcomes were similar, and that the heparin-mediated rhBMP-2-coated bone graft promoted bone regeneration. Histologically, direct ossification findings were observed from the fourth week in the BMP-2/Bio-Oss^®^ group and consolidation was observed on the eighth week. This result concurs with that of a previous study on rhBMP-2-coated bone grafts [22].

The limitations of this study are as follows. First, the concentration of rhBMP-2 added to improve the bone formation was not optimized. Studies disagree on the effect of rhBMP-2 concentration on new bone formation [17,36]. According to one study, different concentrations of rhBMP-2 did not differentially affect bone formation [37], whereas in another study, bone formation increased with rhBMP-2 concentration [17,36]. In view of the different experiment approaches used, it is not possible to determine an appropriate concentration level. Furthermore, the roles of growth factor are dependent on species, the object of the study, the method of application, and the defect type used [38].

## 4. Experimental Section

### 4.1. Immobilization of rhBMP-2 on Bio-Oss^®^

The Bio-Oss^®^ not coated with any material, was used as a positive control. In the BMP-2/Bio-Oss^®^ group, to immobilize rhBMP-2 on the Bio-Oss^®^, Bio-Oss^®^ was coated with heparinized dopamine. Briefly, heparinized dopamine (2 mg/mL) was dissolved in a 10 mM of Tris-HCl (pH 8.0) buffer, 100 mg of Bio-Oss^®^ was added, and exposed under blocked light for 24 h. After the reaction, Bio-Oss^®^ was washed with distilled water, freeze-dried, and then rhBMP-2 expressed by *Escherichia coli* (Cowellmedi, Pusan, Korea) was reconstituted and diluted in a buffer to a concentration of 0.1 mg/mL. Bio-Oss^®^ was loaded with 0.1 mL of rhBMP-2 solution to obtain implanted concentrations of 10.0 μg. In the Bio-Oss^®^ group, Bio-Oss^®^ was loaded with buffer alone.

### 4.2. Assessment of the Morphological Characteristics of Bio-Oss^®^

Bio-Oss^®^ and BMP-2/Bio-Oss^®^ were morphologically analyzed using a scanning electron microscope (SEM; S2300, Hitachi, Tokyo, Japan). Samples were coated with Pt using a sputter coater (Eiko IB, Tokyo, Japan) and examined by SEM. To assess immobilization of rhBMP-2 on the surfaces of the heparinized Bio-Oss^®^, rhBMP-2 was conjugated with GFP and subsequently immobilized on heparinized Bio-Oss^®^. Briefly, heparinized Bio-Oss^®^ was immersed in 0.1 M MES buffer (pH 4.5) and then GFP-conjugated rhBMP-2 was added. GFP-conjugated rhBMP-2 on heparinized Bio-Oss^®^ was observed under a confocal laser scanning microscope (LSM700, Zeiss, Oberkochen, Germany) at 1, 3, 7, and 10 day.

### 4.3. X-ray Photoelectron Microscopy (XPS)

The surface compositions of Bio-Oss^®^, heparinized Bio-Oss^®^, and heparinized rhBMP-2-Bio-Oss^®^ (BMP-2/Bio-Oss^®^ group) were analyzed by XPS on a K-alpha spectrometer (ESCALAB250 XPS System, Theta Probe AR-XPS System, Thermo Fisher Scientific, Glasgow, UK) using an Al Kα X-ray source (1486.6 eV photons) at the Korea Basic Science Institute Busan Center (Busan, Korea). The C1s peak at 284.84 eV was used as a reference for all binding energies. The area of each peak was normalized *versus* total peak area of all elements to calculate surface atomic percentages.

### 4.4. Release Profiles of rhBMP-2

To demonstrate the amount of rhBMP-2 released from BMP-2/Bio-Oss^®^, 30 mg of BMP-2/Bio-Oss^®^ were placed in a 15-mL tube (Falcon, Austin, TX, USA) containing 1 mL PBS (pH 7.4) with gentle shaking at 100 rpm at 37 °C. At predetermined time intervals of 1, 3, 5, and 10 h, and 1, 3, 5, 7, 14, 21, and 28 days, the supernatants were harvested and replaced with fresh PBS solution. The supernatants were frozen at −20 °C to evaluate the amount of rhBMP-2 released. The amount of rhBMP-2 released was evaluated with an enzyme-linked immunosorbent assay (ELISA) kit according to the manufacturer’s instructions using a Flash Multimode Reader (Varioskan™, Thermo Scientific, Waltham, MA, USA) at a wavelength of 450 nm.

### 4.5. Animals and Surgery

**Figure 9:**
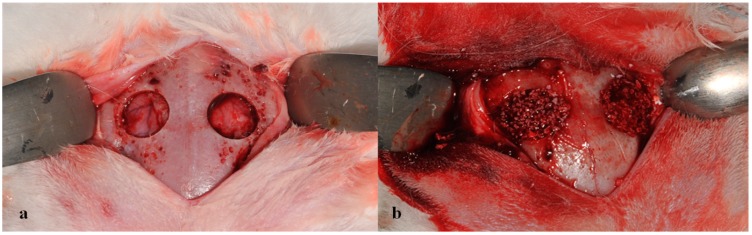
Formation of calvarial defect (8 mm diameter) and implantation of experimental materials. (**a**) two defects were formed with trephine bur; (**b**) defect areas were implant with or without Bio-Oss^®^ or BMP-2/Bio-Oss^®^.

Eighteen 12–13-week-old male New Zealand white rabbits (3.3–3.5 kg) were housed in a light and temperature-controlled environment and given food and water *ad lib*. All animal experiments were performed in accordance with the guidelines issued by the Animal Care and Use Committee of Korea University Guro Hospital (KUIACUC-20120917-2, 20 October 2012). Rabbits were anaesthetized intraperitoneally with ketamine (75 mg/kg) plus xylazine (10 mg/kg). For each rabbit, the dorsal portion of the cranium was shaved aseptically and prepared for surgery. Calvarial defects were produced by first making a sagittal incision of ~20 mm opened over the scalp. The periosteum was removed and a full-thickness bone defect (8 mm in diameter) was trephined in the center of each parietal bone using a slow speed dental drill (Marathon 3, Saeyang Company, Seoul, Korea) without damaging the dura (two implants per calvarium). Bone defects were randomly implanted with Bio-Oss^®^ or BMP-2/Bio-Oss^®^ or left empty (Figure 9). The amount of graft used was based on defect volume, and all groups received the same amount of graft (about 0.1 g). At 4 and 8 weeks after surgery, nine animals in each time point were sacrificed and two samples of calvarial defects were obtained per rabbit, and thus, 36 defects were prepared in total. Animals were allowed to recover for 4 or 8 weeks after surgery, after which they were sacrificed by CO_2_ inhalation. To collect implants, skin was dissected and defect sites were removed with surrounding bone. The biopsied specimens were fixed and prepared for μCT analysis and histomorphometry analysis.

### 4.6. Micro-Computed Tomographic Analysis

At each time point, harvested calvarias were scanned with a μCT scanner (Skyscan 1173; Skyscan, Kontich, Belgium). Calvarial specimens of thickness 0.012 mm were scanned. Digital micro-radiographic images were acquired at 130 kV and 30 μA. Scanned images were reconstructed using NRecon software (version 1.6.3.2, Skyscan). Data were analyzed using a CT analyzer (version 1.11.5.1, Skyscan) and remodeled using Realistic 3D-Visualization (Skyscan). Calibrated 3-D images were shown in the gross profiles of the specimens. Because initial defects were round (diameter 8.0 mm), regions of interest were placed based on initial defect size and shape. Total volumes (%) of newly formed bone within ROIs were measured by assigning a threshold for total bone content (including trabecular and cortical bone ranges) and subtracting any contributions from Bio-Oss^®^.

### 4.7. Histologic Specimen Fabrication and Histometric Measurements

**Figure 10:**
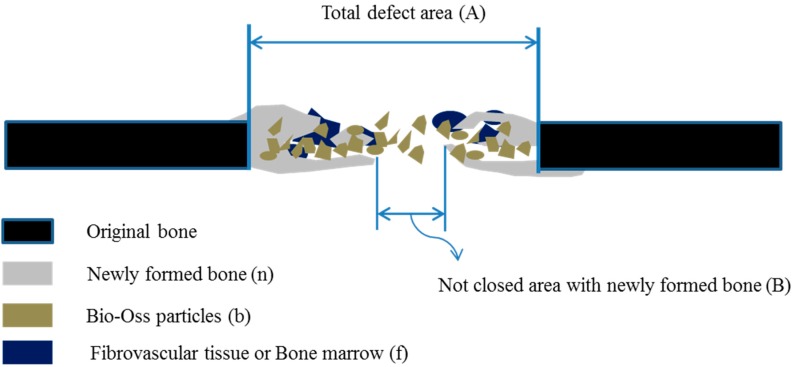
Schematic drawing of a calvarial osteotomy defect Amounts of new bone (%) as a percentage of augmented area = n/(n + b + f) × 100 (where n is newly formed bone, b is Bio-Oss^®^ particle, and f is fibrovascular tissue or bone marrow), Defect closure (%) = (A − B)/A × 100.

Calvarial specimens including implants was prepared after sacrifice, fixed for 2 weeks in neutral buffered formalin solution (Sigma Aldrich, St. Louis, MO, USA), dehydrated using an ethanol series (70%–100%), and embedded in Technovit 7200 resin block (Heraeus KULZER, South Bend, IN, USA). Block of polymerized resin was cut through the center of calvarial defects using an EXAKT diamond cutter (KULZER EXAKT 300, EXAKT, Norderstedt, Germany). Slides mounted with specimens 30 μm thick were prepared from 400 μm-thick sections using an EXAKT grinding machine (KULZER EXAKT 400CS, EXAKT, Norderstedt, Germany). Tissues were stained using hematoxylin-eosin (H&E). After conventional microscopic examination, computer-assisted histometric measurements were made using an automated image-analysis system (Image-Pro Plus, Media Cybernetics, Silver Spring, MD, USA). Histometric parameters were defect closure and new bone rate (Figure 10). Defect closure was defined as the area between the total defect margin and the in-growing bone margin in millimeters. Percent defect closure was obtained by subtracting defect closure from total defect area and expressing this as a percentage of total defect area. The amount of new bone (%) was defined as the area of newly formed mineralized bone (excluding bone marrow and fibrovascular tissue) in the defect as a percentage of the total augmented area.

### 4.8. Statistical Analysis

Statistical analysis was performed using SPSS 15.0 (SPSS, Chicago, IL, USA). Histomorphometric results of calvarial defect samples are expressed as means (±SDs). The analysis was performed using two-way analysis of variance (ANOVA), and Bonferroni’s *post hoc* test was used to determine the significances of intergroup differences, and the Mann-Whitney test was used to determine the significances of differences between samples analyzed at 4 and 8 weeks after surgery.

## 5. Conclusions

We describe a new type of osteoinductive bone matrix comprised of Bio-Oss^®^ and rhBMP-2. In terms of amounts of new bone expressed as percentages of augmented area, the Bio-Oss^®^ and BMP-2/Bio-Oss^®^ groups showed significantly larger areas of new bone than the non-implanted control groups. Furthermore, the heparinized BMP-2/Bio-Oss^®^ group had significantly greater new bone area than the Bio-Oss^®^ group at four weeks after surgery. These findings suggest that heparinized rhBMP-2-Bio-Oss^®^ has excellent bone regeneration enhancing effects in early stage of bone regeneration.
